# Notes and News

**Published:** 1889-09-21

**Authors:** 


### Notes and News.

Collected and Communicated.

There has just been opened at Strathpeffer, in Scotland, a peat bath, such as many invalids have found beneficial at Carlsbad and Marienbad. There are certain medicinal properties in peat which are useful in certain diseases; and seeing how common the peat is on the Highland hills, it is ridiculous that baths have not been established before this. Dr. Fox has done much to develop Strathpeffer Spa, and now the rumour that the Queen thinks of taking the waters there has attracted much attention to this pretty and healthy spot.

The North-West London Hospital in Kentish Town is little known, yet much beloved. It goes quietly on its way, treating 650 in-patients and 43,000 out-patients yearly, and making its accounts balance and pleading no debt. It has eighteen endowed beds, and a useful half-crown fund. We are inclined to think that the sympathy and sustained friendly interest it arouses is largely secured by the fact that it is open to inspection daily between the hours of two and five, and that all visitors are cordially welcomed and shown round the wards.

Public interest is still excited about the new workhouse infirmary at Birmingham, and all are anxious to know how the colossal undertaking will turn out. The infirmary has been built at a cost of £65 per head, which many said could not be done. The total cost of building, fittings, and furniture was £79,479, of which £8,000 went to providing the newest inventions for the laundries, kitchens, etc., and so saving time, and labour, and, in the end, money. There are still lying-in wards, boiler-houses, and other necessaries to be obtained, but the chairman promises that the total expense for everything shall not exceed £90,000.

Mr. Howard Vincent, M.P., made a capital speech at the annual meeting of the Sheffield General Infirmary. Some discussion on the abuse of hospitals commenced the meeting, but Mr. Vincent fought shy of the subject, and merely referred to the work of the infirmary. He said he was sure it would be a source of great satisfaction to the governors of the institution to learn from the report for the past year how considerable had been the diminution in the amount of suffering in the town, as evidenced by the admissions to the infirmary, and more especially would he venture to congratulate the town and the board on the fact that the accidents and emergencies had shown a decrease. There were 868 fewer accidents and emergencies in the past year than in the previous one, and 1,528 less than was chronicled in the report for 1888. That showed that great care had been taken in the management of the large works in the town to prevent by all possible means the occurrence of accidents to those employed in them.

At the Pharmaceutical Conference, Mr. Liebold made a sensation by reading a paper on arsenic in glycerine, which of course, turned all thought towards Mrs. Maybrick. He said he had examined a number of samples of glycerine, and found a large proportion contained arsenic. The quantity of arsenicus acid varied—one in four thousand to one in six thousand—and was very large, but some samples were free from arsenic. The president of the conference said the matter was a serious one, and they should find a source. Dr. Allen said the fact in connection with the recent well-known case was very important. Dr. Thresh agreed that the quantity was large. They must find every way in which such a poison could enter the system. An interesting discussion followed, the matter being regarded as serious.

Business has been brisk in the anthropological section of the British Association. Sir William Turner, the president, delivered the opening address, in which he insisted on the heredity of somatogenic acquired characters. Mr. Francis Galton read two papers on the principles and methods of examinations in physical efficiency. Dr. Wilberforce Smith read a paper on the early failure of pairs of grinding teeth; by Dr. Ridolfo Levi on the development of the wisdom teeth; and by Mr. W. K. Sibley on left-leggedness. Professor D. J. Cunningham dwelt upon the subject of the occasional eighth true rib in man and its relation to right-handedness, and the proportion of bone and cartilage in the lumbersection of the vertebral column in the apes and different races of men, and exhibited models of the head of a man 106 years old, and the head and shoulders of a young orang-outang, both with the brain exposed *in situ*.

Leith Hospital, near Edinburgh, has, within the last few months been extended by the addition of an extra storey to the clinical side, which has just been completed. The latest additions have given two wards to the clinical department with sixteen beds. There have also been added an operating and lecturing theatre, seven bedrooms, and a day-room for the nurses. Like the improvements carried out a few years ago, those just completed have been in the direction of perfecting the sanitary and ventilating arrangements of the hospital, and securing by a mode of treatment hitherto rarely adopted that the walls of the wards shall not become a place of deposit for floating disease germs. The walls of all the wards have been lined with enamelled slate to a height of 4½ feet, the upper portion being treated with Duresco paint, which, after repeated applications, becomes hard and impervious to ordinary atmospheric influences and changes. For the past two years the hospital has been in use as a medical school for women students, clinical teaching being given in surgery and medicine.

Either we are physically degenerating or army recruits are drawn from a worse class than formerly, for the Deputy Surgeon-General's last report is anything but cheerful. It appears that the number of recruits inspected for the regular army at St. George's Barracks, London, during the four years 1885-8 was 23,477, of whom 12,392, or little more than one-half, were passed. London recruiting, of course, embraces men from various parts of the country; but about 63 per cent. are found to belong to the four home counties. The unskilled class of carmen, carters, and van-porters, who supply many good recruits in London, are described as well developed and able-bodied. The "trades" class, such as shoemakers, tailors, bakers, and printers, supply about twenty per cent., while the mechanics count for about fifteen. The clerks, shopkeepers, and professional classes are said to give a large proportion of very fine recruits, especially for the cavalry. Yet one would think the professional classes might do better than become billets for bullets.

The following vacancies are announced this week, the amount of the salary in each case being given after the name of the institution :

**Resident Medical Officers.**

Boscombe Hospital and Provident Dispensary. Resident Medical Officer.—£60, board, &c.
City of London Hospital for Diseases of the Chest, Victoria Park, E. House Physician.—Board, no salary.
Evelina Hospital, Southwark Bridge-road, S.E. House Surgeon.—£70, board, &c.
Female Lock Hospital, Harrow-road, W. Assistant House Surgeon.—Board, no salary.
Flintshire Dispensary, Holywell. House Surgeon.—£100.
General Hospital, Birmingham. Resident Registrar and Pathologist.—£100, board, &c.
General Hospital, Birmingham. Assistant House Surgeon. Board, no salary.
Great Northern Central Hospital, Holloway-road, N. House Surgeon.—£80, board, &c.
Leicester Infirmary and Fever House. House Surgeon.—£120, rising to £160, board, &c.
Northumberland County Asylum, Morpeth. Clinical Assistant.—Board, no salary.
Nottingham General Hospital. Resident Surgical Assistant.—Board, no salary.
Pendleton Branch Dispensary. House Surgeon.—£80, board, &c.
Queen Charlotte's Hospital, Marylebone, N.W. House Surgeon.—£60.
Royal National Hospital for Consumption, Ventnor. Assistant Medical Officer.—£60, board, &c.
South Devon and Cornwall Hospital, Plymouth. House Surgeon.—£100, board, &c.

**Non-Resident Medical Officers.**

Royal College of Surgery, Lincoln's-inn-fields. Examiner in Dental Surgery.

St. Pancras and Northern Dispensary, 126, Euston-road. Honorary Physician.

---

At the half-yearly meeting of Wolverhampton General Hospital Committee, on the motion of the chairman, Dr. M'Munn was appointed honorary pathological officer to the institution. Dr. Millington, in seconding the motion, referred to Dr. M'Munn's microscopical and philosophical researches, and stated that the Royal Society were so impressed with the work of Dr. M'Munn that they had granted him a sum of money for the purchase of books and instruments, in order that his investigations might be prosecuted with advantage.

---

The experts have issued their report on the Huddersfield Hospital question, and it concludes as follows : "Finally, on carefully balancing in our minds the comparative advantages of the two sites, and on taking into full consideration all the arguments which have been laid before us, we feel it to be our duty to advise the Corporation of Huddersfield that, in our judgment, it would be to the permanent advantage of the borough to transfer their hospitals for infectious diseases to the Mill Hill site, even in the event of the Corporation being able to arrange for the purchase of one or two acres of additional land at Birkby."

---

In spite of Whitechapel murders in the very region of Toynbee Hall, Oxford is still bent on conquering brutality, poverty, and vice, by inculcating culture and opening art exhibitions. A new Oxford mission is being established in London, this time in Battersea, and by University College. Its centre will be "University College House." At all these missions a large number of undergraduates spend part of their vacations, in turn assisting the regular workers. The system is that each visitor pays a sum of about thirty shillings a week for board and lodging, and takes part in the usual work. We doubt if these missions do not do as much good work in enlarging the minds and sympathies of the undergraduates as in helping the poor.

The late Mr. Wm. Stirling, of Glasgow, who died a week ago, has left £20,000 to Glasgow charities. The three leading infirmaries get £5,000 each ; the Glasgow Benevolent Association £2,000 ; and Indigent Gentlewoman's Fund £1,000 ; and other charities get £500 each. The deceased was a Turkey-red dyer.

---

For ten long years have bazaars been held at Felixstowe, in hopes of raising enough money to rebuild the women's wards of the Convalescent Home. Early in this month the annual bazaar was held, and on that very day an offer was received from an anonymous friend to give the required £2,000. The bazaar only produced £100—not bad for Felixstowe—but the hearts of all were gladdened by the generous gift to a deserving institution. The present female ward is little better than a large wooden hut.

---

Last week a meeting of ladies and gentlemen was held in Montrose, when a committee was nominated to collect subscriptions in aid of Edinburgh Royal Infirmary. It appears that for the last five years Forfarshire has sent 887 patients to the institution, costing about £4,500, while the only collection made during these years was in Aberlemno Church, which amounted to £2. Provost Scott, Montrose, has written to all the chief magistrates in the county, calling upon them to remove this "slur upon the county."

---

No wonder typhoid is prevalent at Melbourne ! There are said to be two typhoid germs in every three drops of the drinking water supplied to that town. The germ of typhoid fever (*Typhi abdominalis*) forms very striking colonies when grown on gelatine ; their periphery is irregular and transparent, and their appearance resembles mother-of-pearl. Examined under the microscope with a low power, the colonies resemble the circumvolution of the intestines ; their colour in the central part is yellowish-green, and they do not liquefy gelatine. The final test to identify them is to sow them on slices of sterilised potatoes. They seem to make on them no visible impression. Scarcely does the surface of the potatoes become more brilliant and moist, although their surface is literally covered with the bacilli, as shown by the microscope. Their appearance under a high-power microscope is that of a short and thin bacillus, their length averaging between two and three micro millimetres (3-25,000 of an inch). Their movements in the liquids are peculiar and often very rapid. Nothing is more fascinating than the search for and culture of these germs.

---

The working-men of Hull have risen to the occasion, and devised a scheme by which, we do not doubt, they will rescue their infirmary from debt and distress. At a crowded meeting held last week Mr. T. E. Wing read a paper on the subject. Having pointed out the present position of the infirmary finances, and the results that would follow if an effort were not made, Mr. Wing said that after much and long consideration of the matter he had hit upon a scheme, to which, however, he was not wedded, which, if it could be carried out, would raise this sum. He proposed to raise £1,000 by means of a large scroll, containing 20,000 names, each contributing one shilling. This "magnificent roll-call of mercy" might be composed of 1,000 sheets of ruled foolscap, with spaces for number, name, occupation, and contribution, and he would put twenty names on each sheet, which they might call "The Infirmary Samaritan Roll-Call of Helpers to the Helpless." He would ask all classes to contribute. If they fixed the contribution at one shilling, no more and no less, he thought it was possible to get 20,000 people to contribute. The management of the institution was carried on economically, and if they went into the matter with zest and zeal everything was possible to them. The meeting approved of Mr. Wing's scheme, which will be carried out.

